# SARS-CoV-2 and Endothelial Cells: Vascular Changes, Intussusceptive Microvascular Growth and Novel Therapeutic Windows

**DOI:** 10.3390/biomedicines10092242

**Published:** 2022-09-09

**Authors:** Antonio Giovanni Solimando, Donatello Marziliano, Domenico Ribatti

**Affiliations:** 1 Guido Baccelli Unit of Internal Medicine, Department of Biomedical Sciences and Human Oncology, School of Medicine, Aldo Moro University of Bari, 70124 Bari, Italy; 2 Department of Basic Medical Sciences, Neurosciences, and Sensory Organs, University of Bari Medical School, 70124 Bari, Italy

**Keywords:** angiogenesis, infections, SARS-CoV-2, COVID-19, intussusceptive microvascular growth

## Abstract

Endothelial activation in infectious diseases plays a crucial role in understanding and predicting the outcomes and future treatments of several clinical conditions. COVID-19 is no exception. Moving from basic principles to novel approaches, an evolving view of endothelial activation provides insights into a better knowledge of the upstream actors in COVID-19 as a crucial future direction for managing SARS-CoV-2 and other infections. Assessing the function of resting and damaged endothelial cells in infection, particularly in COVID-19, five critical processes emerged controlling thrombo-resistance: vascular integrity, blood flow regulation, immune cell trafficking, angiogenesis and intussusceptive microvascular growth. Endothelial cell injury is associated with thrombosis, increased vessel contraction and a crucial phenomenon identified as intussusceptive microvascular growth, an unprecedented event of vessel splitting into two lumens through the integration of circulating pro-angiogenic cells. An essential awareness of endothelial cells and their phenotypic changes in COVID-19 inflammation is pivotal to understanding the vascular biology of infections and may offer crucial new therapeutic windows.

## 1. Introduction

A good understanding of endothelial activation in infectious diseases lays the foundation for improving current approaches and predicting clinical outcomes and future treatments of several clinical conditions. Pathophysiological changes seem to stem from endothelial activation and may provide further enlightenment into SARS-CoV-2 infection management. Thromboresistance, vascular integrity, angiogenesis, vascular remodeling, the regulation of blood flow and the control of immune cell trafficking are essential processes, and the disruption of endothelial processes entails vascular dysregulation in COVID-19.

SARS-CoV-2 infection induces vascular endothelial cell damage [1] while exerting pleiotropic systemic effects [2,3,4]. The COVID-19 virus causes damage not only to respiratory epithelial cells but also to several other extra-respiratory targets [5]. Indeed, the angiotensin-converting enzyme 2 (ACE2) receptor is widely expressed by several tissues and is a binding site for the spike protein [6]. Extra respiratory involvement is uncommon [7,8,9,10], although cardiovascular effects are common, including arrhythmias, heart failure and myocarditis [11,12,13]. Linder et al. quantified the virus copy number in the heart while correlating the gene expression profile of pro-inflammatory cytokines [13]. The olfactory system is no exception since an intense vasculature surrounds the epithelial and neuronal components [14]. Looking at the ACE2 receptor in the olfactory bulb, the investigators found the highest receptor concentration in both vascular endothelial cells and pericytes [14]. Collectively, the evidence points towards vascular endothelialitis, affecting the lung, the cardiovascular system, the brain, and the olfactory organ [15,16,17]. Vascular events such as microthrombi and intussusceptive microvascular growth can develop from a systemic activation of inflammatory cascades mediated by massive cytokines release [18].

Further efforts are needed to fully elucidate whether endothelialitis is exclusively secondary rather than directly caused by the infection [19]. The endothelium is a distributed organ with fundamental properties, being one of the most abundant cell types. It constitutes up to one kg in mass and comprises at least one trillion cells [20]. The surface area can cover up to four thousand m^2^, mostly made up of capillaries intimately associated with blood. The endothelium is morphologically and phenotypically heterogeneous, allowing specialized functions in benign and malignant conditions [20,21,22]. Several highly coordinated endothelial cell (EC) functions have been described. Firstly, it forms a conduit for blood and nutrients, with crucial functions for tissue survival and critical features in angiogenesis [23,24]. In addition, endothelial cells also establish a selective barrier, allowing specific molecules and fluid constituents to leave the extra-vascular space and enter the intravascular compartment [25].

Moreover, EC regulates vascular tone by releasing specific mediators involved in inflammatory responses and modulating hemostasis [26,27]. Any derangement of these mechanisms leads to severe barrier dysfunction and failure of physiologic processes [28,29,30]. Viral entry into endothelial cells is no longer debated because clear evidence proves that low amounts of virus achieve access to endothelial cells. However, this does not lead to active replication as viral RNA is not detected [31]. According to current knowledge, secondary mechanisms for endothelialitis may be implicated rather than direct viral infection and direct toxicity. Endothelial cells may be activated by factors released by platelets in the same way as during hypoxia. This leads to VEGF secretion, which causes endothelial activation and angiogenesis.

Furthermore, even resting endothelial cells are responsible for several vascular homeostatic processes. Firstly, thromboresistance is accomplished to some extent by the presence of natural anticoagulants, including tissue factor pathway inhibitor (TFPI), antithrombin (AT), and thrombomodulin [32,33,34]. Secondly, platelet modulators expressed by endothelial cells contribute to thromboresistance via ADP/ATPase on the endothelial cell surface, inhibiting platelet aggregation, the production of nitric oxide (NO) through the action of NO synthase 3 (NOS3) and prostacyclin-2 (PGI-2) via cyclooxygenase (COX) [32,33,34,35]. Endothelial cells are also critical for vascular integrity and are actively involved in angiogenesis via the inter-endothelial cell junctions [36,37,38]. Thirdly, the endothelial production of NO and other mediators strictly regulate blood flow [32,39]. In addition, the vascular endothelium oversees white blood cell trafficking from the intra- to the extra-vascular space thanks to adhesion molecules. Finally, it acts as a gatekeeper for the immune and inflammatory system, as it can open intercellular junctions [21,40,41,42]. Blood vessels and their phenotypic alterations in SARS-CoV-2 infection represent an archetype model for severe infections involving the endothelial interface [33,43], offering loopholes for novel targeting in systemic inflammation. 

## 2. Loss of Thrombo-Resistance in COVID-19

The high risk of thrombosis in COVID-19 pinpoints the pivotal role played by endothelial damage in targeting vascular beds of all sizes. Although several attempts to stratify the risk of disease severity and progression [44,45] and significant improvements in early interventions with the various pharmacological approaches have been made [46,47,48], in its severe phenotype COVID-19 remains a significant challenge for clinicians [49]. Numerous insights focused on vascular biology, infection and coagulation have helped to develop fascinating hypotheses and uncovered antiphospholipid antibody as a natural antimicrobial response, sometimes leading to antiphospholipid (aPL) syndrome (APS) [50,51]. While COVID-19 and APS are different conditions, they share some fundamental similarities which define common vascular disease pathobiology, leading to strokes [51] and cardiovascular ischemic events [52,53,54]. It was mainly challenging to demonstrate that antiphospholipid antibodies tracked close to large vessels’ thrombotic events, but microvessels are often involved [52,53,54]. Autoimmunity and vascular antigen are other examples of immune dysregulation in COVID-19, often affecting blood components [55]. Undeniably, B-lymphocytes-derived molecular mediators, involved in the extrafollicular B cell response, appear to bypass regular germinal center checkpoints in COVID-19 [56]. In addition, some of these autoantibodies may have deleterious functions, as anti-interferon (IFN) antibodies seem to disrupt IFN-mediated antiviral responses [57]. Jason Knight’s group revealed that prothrombotic autoantibodies in serum from hospitalized patients suffering from COVID-19 were correlated with clinical biomarkers and respiratory status [58]. Using an elegant in vivo approach, researchers isolated the whole IgG fraction from patients and were able to trigger thrombosis with COVID-19 IgG to the same extent as antiphospholipid antibody does in mice with an electrolysis-activated endothelium [58]. Hollerbach et al. described how IgG fraction transfer in mice, leading to accelerated thrombosis and that lipid-binding aPL, targeting cell surface lysobisphosphatidic acid complexed with the endothelial protein C receptor (EPCR), plays a novel role in mediating thrombosis [59]. These insights into vascular biology and prothrombotic state highlight the key roles of endothelial cells, platelets, neutrophil extracellular traps (NETs) and complement in mediating vascular damage in COVID-19.

Thrombosis and coagulopathy have recently emerged as severe consequences of SARS-CoV-2 infection [60]. Altered fibrin homeostasis and angiotensin signaling entail increased thrombin and purinergic receptors activity, leading to platelet activation and other effector mechanisms [61]. This further exacerbates inflammation and fuels tissue injury [62]. The lungs would be the main target of such a mechanism, although systemic effects may follow. Endothelial cell activation and thrombosis are two closely related processes. Angiotensin signaling dysregulation worsens thrombosis and increases tissue injury [63].

Furthermore, it enhances proteinase-activated receptor (PAR) signaling [63], tissue factors synthesis and expression in several cell types, increased cleavage of circulating prothrombin into thrombin and increased purinergic signaling [64]. Additionally, megakaryopoiesis and complement activation mediated by SARS-CoV-2 seem to lay the foundations for the onset and progression of thrombo-inflammation in COVID-19 [65]. This ultimately leads to EC activation [66]. These findings have unraveled new pathways that may identify platelet and clotting cascade inhibitors as novel therapeutic targets [42,67].

## 3. Endothelial Cells, Platelets, and NETs in COVID-19

Early in the pandemic, Libby and Lüscher emphasized the role of endothelial dysfunction in COVID-19, predisposing to a prothrombotic state [68]. Next, endothelial cell infection was described [69]. Nonetheless, there were significant drawbacks to translating autoptic evidence into an in vitro model that hinders a comprehensive disentanglement of the underlying mechanisms. However, endothelial damage and fibrin microthrombi, paralleling endothelial cell derangement via intussusceptive microvascular growth (IMG) [5], take place. Endothelial glycocalyx represents an active layer of molecules which contributes to lung homeostasis and prevents lung injury and is therefore a candidate for novel therapeutic windows. First, glycocalyx is one of the major components of the endothelial cell barrier which prevents fluid and several proteins and albumin leakage into the alveolar compartment [70,71]. Thus, pathological shedding of transmembrane proteins is involved in endothelial cell glycocalyx damage [70], as demonstrated by enhanced levels of sTie2 and Syndecan-1 in COVID-19. Accordingly, Schmaier et al. underlined endothelial activation biomarkers, such as von Willebrand factor (vWF), E-selectin and Tie2 levels, correlated with COVID-19 severity [71]. However, despite clear evidence regarding endothelial cell damage, the mechanisms underlying direct infection and cell activation remain unclear. Nonetheless, glycocalyx damage prompted intense investigation to envision novel therapeutic windows [70,71].

Manne et al. showed that high levels of P-selectin correlated to platelet activation ex vivo when comparing COVID-19 patients with controls. Leukocyte/platelet aggregates were also detected as secondary platelet activation markers [72]. Moreover, platelets promote thrombo-inflammation in SARS-CoV-2 pneumonia, a potential source of cytokine release and endothelial cell activation [73]. Therefore, platelets seem to be activated in COVID-19 while forming aggregates with leukocytes, although this event seems hard to be predicted, despite disease severity.

Brinkmann et al. showed that NETs actively contribute to microbial killing [74]. Indeed, NETs are extracellular spider webs made of antimicrobial proteins that kill microbes, backbone DNA and other proteins from the nucleus (i.e., histones). Using discarded serum from clinical biochemistry laboratory tests, Zuo et al. demonstrated that cell-free DNA, MPO–DNA complex, and citrullinated histone H3 levels significantly exceeded the concentrations measured in controls [75], thus showing NETs to be correlated with clinical biomarkers and respiratory status. Likewise, Middleton et al. emphasized that COVID-19 severity parallels NETs and platelets interaction in the lungs of subjects who died of COVID-19 [76]. Skendros et al. described NETs from COVID-19 to be particularly enriched with tissue factors after culturing platelet-rich plasma with healthy neutrophils and measuring thrombin-antithrombin complexes [77].

Additionally, Zuo et al. found that autoantibodies stabilize neutrophil extracellular traps in COVID-19 [75]. Collectively, NETs parallel disease severity, most likely contributing to thrombosis. Hence, they can potentially be pharmacologically targeted. Holter et al. confirmed these findings, substantially extending the correlation of complement and correlating complement products with a worse oxygen status [78]. 

Magro et al. reported C5b-9 in the microvasculature of interalveolar septa [79], pioneering the concept of systemic complement activation in COVID-19. Remarkably, complement and endothelial activation seem to coexist, suggesting an enhanced complement activation, most likely contributing to endothelial damage. This potentially underlines a novel therapy target for COVID-19 endothelialitis [42,80,81]. 

## 4. Pathobiology of COVID-19: Lung Imaging Foreshadows Vascular Changes and Endothelialitis

Since the beginning of the COVID-19 pandemic, a clear pattern of lung changes has been highlighted on chest X-ray and computed tomography (CT), characterized by typical features of bilateral lower lobules and peripheral lung shadowing. This suggests a diagnosis associated with distinctive clinical features [82,83,84]. Although radiological severity at admission does not correlate with clinical outcome, systems for classifying severity have been developed [85]. Initially, CT was used for diagnosis in China when PCR testing was unavailable. It showed a high sensitivity [86], albeit non-specific and affected by several caveats. Remarkably, many typical features conventionally associated with viral or inflammatory pneumonia were lacking.

Nevertheless, viral entry into the respiratory tract and subsequent pneumonia were detected, often worsening to thromboembolism and vessel damage requiring timely treatment for aggressive COVID-19. However, this failed to disentangle the mechanism underlying the SARS-CoV-2 distinct pattern of lung and cardiovascular system changes [35]. Histologic studies boosted our insight into the complex underlying pathobiology, revealing several vascular features to be relevant, as well as respiratory findings [87]. Besides airway inflammation, consolidation and diffuse alveolar damage, lung involvement seems to be characterized by macro-thrombosis, vascular dilatation, microvascular angiopathy with micro-thrombosis, and pulmonary hemorrhage, capillaritis and endothelialitis [88]. Histology has been limited to autoptic studies. Therefore, no direct correlation of post-mortem tissue with pre-mortem CT has been shown, often due to a lack of availability of in vivo lung biopsies [89]

The edematous, infected lung does not differ from that in classical acute respiratory distress syndrome (ARDS), although atypical findings for a conventional ARDS have been described, such as swollen endothelial cells engorged within the capillary network. Moreover, SARS-CoV-2 was shown by electron microscopy to infect the vascular endothelial cells [90,91]. Additionally, the normal lung and the COVID-19 infected lung were compared using the vascular corrosion casting technique, showing evident architectural destruction in both the pulmonary epithelium and endothelium [5].

Initial evidence hints at endothelial dysfunction in SARS-CoV-2 infection, playing a crucial role in severe COVID-19 pathogenesis [31]. Debated evidence points toward complex viral pathobiology involving the vessel wall. Surprisingly, SARS-CoV-2 may enter endothelial cells only at low levels, and there are no precise data regarding the viral ability to replicate therein. Conversely, COVID-19 is undeniably associated with vascular inflammation, vascular events leading to fibrin microthrombi formation, intussusceptive microvascular growth and disseminated intravascular coagulopathy (DIC). In addition, vascular endothelialitis has been reported. However, evidence to date does not support direct endothelial cell dysfunction determined solely by SARS-CoV-2. Unquestionably, according to several in vitro studies, endothelial cells taken from different tissues show a low expression of the ACE2 receptor, and no TMPRSS2 mRNA has been detected. Thus, aside from coronary artery endothelial cells, other endothelial cells showed low rates of infection; even when present, replication does not occur, as viral RNA has not been detected. Furthermore, TNF-α exposure did not increase spike protein detection, which is confined to the endosomal compartment [31]. Endothelial cells can sense an infection in neighbouring lung alveolar cells and help to mount a pro-inflammatory response, although they are rarely directly affected by SARS-CoV-2. Moreover, the vessel wall can be indirectly impaired by the response of pericytes to infection. As a result, endothelialitis and endothelial dysfunction may lead to apoptosis, as demonstrated by Cas3 detection, apical extrusion, or the secretion of cytokines such as IL-6 and CXCL10 and induction of ICAM1 expression [92]. Additionally, elevated angiogenic factors levels have been observed, even though it is still debated whether this could be attributed to the related hypoxia due to lung damage [93]. A paradigmatic example of the pleiotropic, dynamic, and context-dependent effect of SARS-CoV-2 infection was illustrated when investigating the CNS vascular endothelium. The brain and the olfactory tubercle seem not to be affected by SARS-CoV-2 infection due to a low expression of the ACE2 receptor on the leptomeninges, even though anosmia is frequently reported as a presenting symptom. Indeed, no specific histopathological finding, such as fibrinoid necrosis and intraparenchymal hemorrhages, has been described in SARS-CoV-2 infected post–mortem specimens. Conversely, many cases show cerebrovascular abnormalities such as ischemic stroke, reflecting altered general coagulation homeostasis and secondary endothelial involvement due to systemic disease [94]. 

Contrariwise, hypoxia, leading to VEGF release, causes endothelial activation [95]. There are many ways in which endothelialitis can be present without significant infection of endothelial cells. Therefore, these findings point towards a multifaceted SARS-CoV-2 role in driving direct endothelial cell toxicity.

It is widely recognized that endothelial cells are crucial to vessel homeostasis, and any alteration can trigger several pathologies. Oxidative stress is the ultimate result of most mechanisms leading to endothelial inflammation and dysfunction. Tobacco and electronic cigarette smoking and hyperglycemia in diabetes may lead to altered NO availability and excessive ROS production. Conversely, hypertension causes increased NADPH oxidase, ADMA, endothelin-1 and angiotensin-2, inducing ROS overexpression. Additionally, hypercholesterolemia causes an endothelial eNOS and iNOS imbalance due to high amounts of oxidized LDL [96]. 

Furthermore, oscillatory shear stress leads to pro-inflammatory endothelial cell activation, associated with increased monocyte adhesion and endothelial cell apoptosis. Inflammatory mechanisms contributing to endothelial dysfunction, such as NETs and NLRP3 inflammasomes, have been recently implicated and are currently under investigation. Excessive NETs cause vascular leakage and complement activation, leading to endothelial injury and inflammation. Lastly, the massive release of inflammatory cytokines results in increased oxidative stress and dyslipidemia in chronic autoimmune inflammatory diseases. However, the role of autoantibodies as indicators of endothelial dysfunction remains to be elucidated [96]. As a result of SARS-CoV-2 infection, the pathobiological cascade shifts towards thrombosis, as micro thrombosis is more likely to develop upon blood vessel damage [34]. This phenotype becomes generalized throughout the body [97]. SARS-CoV-2 infection drives a respiratory involvement that significantly differs from Influenza-related pneumonia due to IMG [5]. Remarkably, both IMG features and sprouting angiogenesis correlate with hospitalization duration [5,98].

## 5. Loss of Vascular Integrity: IMG in COVID-19

IMG is crucial in vascular injury, marking a critical reaction to endothelial damage. A vascular tree stems from a capillary plexus in early embryonic development due to intussusceptive pillar development and pillar fusions, a process named “intussusceptive arborization” [99]. This initial assembly of progenitor and endothelial cells form blood islands that build a pattern of primitive circulation [99]. The blood’s primitive structures collapse. Drop-down pillars can enter columns from the bottom and eventually create holes that separate and spread out into tubes [99]. Thus, from a flat two-dimensional cell surface, a complex architectural division takes place and shapes the blood vessels, as demonstrated by the chorioallantoic membrane (CAM) assay [22,99], a classic angiogenic embryological model employed in physiology, oncological and non-oncological investigation [29,38]. IMG is also present in mice, as demonstrated in the development of the choroid plexus in the eye [100,101]. IMG drives laser-induced choroidal neovascularization in adult mice upon laser-induced intussusception injury as a response to a damaging stimulus [102]. This process occurs within seconds to minutes as a response to the initial injury [103]. 

Thus, it is tempting to speculate that IMG may be of the utmost importance in COVID-19, considered a blood vessel disease [104], thanks to intriguing preliminary evidence of IMG involvement in the CNS, as shown in both non-malignant and malignant conditions [105,106]. The vasculature may not be the only connection between the eye and CNS, but may instead be a sign of multisystem involvement, as demonstrated by an extensive study of 11,116 subjects hospitalized for COVID-19. Indeed, looking at comorbidities such as age, obesity, and diabetes, Ramiali et al. described age-related macular degeneration in 20% of cases, entailing a 3-fold higher mortality risk [107]. Thus, the multidisciplinary nature of this inquiry highlighted that patients with vascular involvement were more likely to be intubated and die [107]. These compelling data underline a link between infection, inflammation, vascular system and complement activity that have prompted the ongoing research on angiogenesis in COVID-19. The levels of angiogenesis markers in COVID-19 patients were associated with the presence of IMG in the lung tissue of COVID-19 patients [5]. A wound healing process follows the vascular damage due to the microvascular infection of the SARS-CoV-2. Pro-angiogenic stimuli are released as a microcirculation response, and thrombosis ensues [108].

Nevertheless, a unique form of IMG takes place rather than simple sprouting. A single capillary winds up due to micro thrombosis in the attempt to create more blood vessels. Despite its collapse, a seeding of endothelial cells creates new walls split into multiple vascular channels [109,110,111,112,113]. When comparing IMG with sprouting angiogenesis in COVID-19, a statistically significant difference was detected between COVID-19 vs. H1N1-affected lungs, although this was less pronounced than in IMG [5]. From a pathobiological standpoint, IMG indicates a normal wound healing response [114], owing to the coagulation cascade. To bypass the thrombosis, the endothelial cells seem to fuel collateral circuits until classical sprouting angiogenesis can intervene [115]. Therefore, hypoxia is related to microthrombi, prime vessel dilation, incorporation, and expansion by circulating progenitor cells, increased nitric oxide, vasodilation, and vascular endothelial growth factor (VEGF) production [116]. The gene array from the COVID-19 infected tissues was compared to Influenza A-affected organs by Ackermann et al., who investigated the overlapping of those genes. In the context of the extreme cytokine storm, a well-known factor of disease severity in COVID-19 [42,45], VEGF-A is deemed instrumental in lungs infected both by H1N1 and by SARS-CoV-2 [5].

## 6. Angiogenic Factors Involved in COVID-19

### 6.1. VEGF

**Figure 1:**
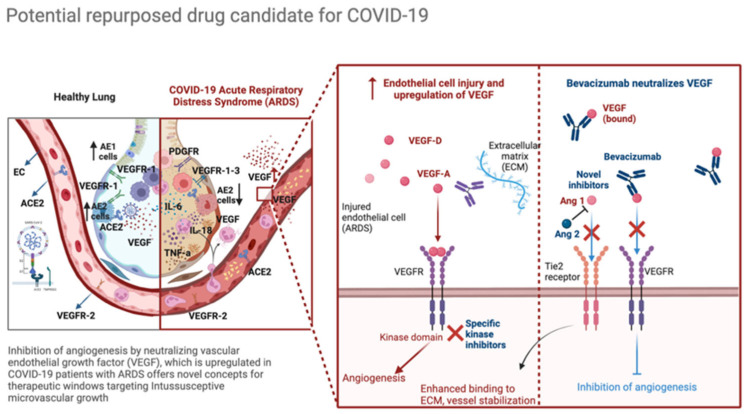
Potential repurposed drug candidate for COVID-19. Modulation of VEGF and angiogenesis hold the potential to slope the equilibrium between vascular homeostasis and viral-related damage. Bevacizumab has been used in ongoing clinical trials in critically ill patients with severe COVID-19 [107,119]. Knowledge of the molecular events governing tumour angiogenesis and intussusceptive microvascular growth has promoted several therapeutic strategies to block dysfunctional endothelial cells in COVID-19. Successful therapeutic targeting of VEGF and its receptors is the most promising strategy for the future. Endothelial cells are characterized by context-dependent and dynamic cell motility and interactions with soluble factors. Peptides derived from normal proteins by proteolytic cleavage, including endostatin and tumstatin, inhibit angiogenesis through mechanisms that include interfere with integrin function. Aberrant cell signaling-directed approaches and the inhibition of epidermal growth factor (EGF)-family receptors, whose signaling activity is upregulated in damaged endothelial cells, result in downregulation of VEGF and IL-8, while increasing the expression of the antiangiogenic protein thrombospondin-1. Ras/MAPK, PI3K/Akt, and Src kinase signaling shape cell proliferation and EC homeostasis, offering a crucial field of investigation [14,107,119] of alveolar type 1 (AE1) and type 2 (AE2) cells. Figure created with BioRender.com (accessed on 9 June 2022).

COVID-19-affected lungs tend to develop angiogenesis, and VEGF, a key molecule in vascular biology and pathology [117,118], plays a significant role in this scenario and vascular pathobiology of infections [5]. Following SARS-CoV-2-induced ACE2 suppression, the VEGF-VEGFR system is deranged, and its modulatory effect on VEGF activation is hampered. VEGF displays a critical function in the pathogenesis of COVID-19, being correlated to pulmonary edema, declining oxygen saturation, and vascular remodeling. Indeed, disturbing alveolar-capillary membrane integrity, leads to fibrin deposition and the development of the acute respiratory distress syndrome (ARDS)-related fibro-proliferative phase (Figure 1).

Disease severity has been correlated to levels of VEGF-D detected in plasma [117], as VEGF-D facilitates SARS-CoV-2 lung-to-blood transmission. Despite no consistent evidence confirming these hypotheses, COVID-19 causes neuroinflammation that disrupts the BBB. No clear link between the VEGF-A pathway and neuro-inflammation in the brain has been established, although COVID-19 patients develop inflammatory responses which can trigger a disruptive effect on the blood–brain barrier, thanks to VEGF-A and other mechanisms. Statistically powered confirmatory investigations are needed to support these hypotheses further and generate preliminary data [118]. Indeed, studies focusing on retinal involvement documented infection and pathological involvement in COVID-19 of the retina and central nervous system [107,119]. The blood–retinal and blood–CSF barriers are two examples of boundaries separating privileged anatomical sites in which IMG could play a significant role during SARS-CoV-2 infection [120]. From this standpoint, the retina and the retinal circulation might be a privileged spot that can prompt further research into how best to protect the rest of the body. Alveolar epithelial type 2 (AE2) cells are a significant source of VEGF in adults [121]. VEGF-A is over-expressed in the lung tissue of non-survivor patients [5]. Disease transmission in asymptomatic subjects may be affected by manipulating the VEGF-A165a subtype/b1 domain of neuropilin-1 (NRP-1) signaling, which is upregulated at the transcriptional level in COVID-19 [122]. Several investigators found elevated plasma levels of VEGF-A in sera of COVID-19 patients, which were correlated with disease severity [98,100,123]. Soluble levels of Flt-1 (sFlt-1), a circulating truncated form of the VEGF-A receptor-1 (VEGFR-1/Flt-1), were markedly increased in COVID-19 patients and paralleled disease severity [124,125]. VEGF-D, which stimulates angiogenesis and lymphangiogenesis, was lower in COVID-19 patients than healthy controls [124,125]. Conversely, elevated VEGF-D levels were identified as the most important indicators of disease severity in COVID-19 progression [117].

### 6.2. Other Angiogenic Factors and Therapeutic Windows

Patients with severe COVID-19 exhibited elevated levels of interleukin-6 (IL-6), a VEGF synthesis inducer [126]. IL-6 is involved in tumour angiogenesis and IL-6 levels are directly correlated with VEGF levels [127]. Siltuximab prevents the binding of IL-6 to its soluble and membrane receptors and exhibits an anti-VEGF property [128]. Several investigators found elevated plasma levels of placental growth factor (PlGF) and fibroblast growth factor-2 (FGF-2) in sera from COVID-19 patients correlated with disease severity [123,124,125]. Angiopoietin-2 (Ang-2) is a marker of endothelial activation and a good predictive factor for intensive care unit admission of COVID-19 patients [129]. The expanding clinical scenario triggered by angiogenesis and intussusceptive microvascular growth provides novel insights that may offer hope in several conditions driving ARDS and endothelialitis (Table 1). 

Successful targeting of angiogenesis and intussusceptive microvascular growth has led to an increased interest in developing compounds that target specific steps in the angiogenic process (Table 1) [130]. However, as already demonstrated in other clinical landscapes [131,132,133,134], it is unlikely that vessel-directed approaches will suffice for all SARS-CoV-2 and septicemia-driven conditions [135]. Many trials developed in both malignant and non-malignant conditions have raised awareness of a possible advantage in blocking specific IMG steps (e.g., VEGF). However, several trials will be necessary to confirm the effectiveness of one or a combination of angiogenesis-targeting drugs in different scenarios (i.e., sepsis).

The disruption of glycocalyx and the lack of this protective mechanism leads to alveolar edema due to increased fluid leakage and decreased oncotic vascular pressure. Secondly, glycocalyx coating actively senses shear stress and regulates endothelial NO production accordingly. Hence, any derangement may lead to altered vascular tone and microcirculation, ultimately resulting in altered vessel tone and increased thrombogenicity. Finally, disruption of the glycocalyx layer may further expose leukocyte adhesion molecules regulating WBCs adhesion and rolling. Glycocalyx is mostly made up of heparan sulphate (HS) and several other glycosaminoglycans [136]. The HS degradation by increased pulmonary heparinase activity leads to lung injury in septic patients by unveiling leukocyte adhesion molecules ligands and alveolar extravasation ultimately worsening into ARDS [137]. Furthermore, glycocalyx provides an antithrombotic surface by establishing antithrombin-HS bonds. As heparinase activity increases in critically ill COVID-19 patients, glycocalyx homeostasis is impaired leading to thrombi formation [138]. As mentioned above, the role of Ang-2 in endothelial activation and glycocalyx degradation has recently been investigated [139]. As a response to inflammatory stimuli, endothelial cells secrete Ang-2. By inhibiting the signaling induced by angiopoietin 1 (Ang-1) activating its receptor TIE2, Ang-2 prevents anti-inflammatory signaling normally induced by Ang-1 [139]. The findings of Lukasz et al. determined that Ang-2 treatment rapidly degrades endothelial glycocalyx both in vivo and in vitro, using human umbilical vein endothelial cells and mice [139]. Additional compelling evidence pinpoints Ang-2 inhibition and TIE2 activation to be potential therapeutic targets in sepsis by reducing endothelial glycocalyx shed and improving survival among mice with sepsis [140]. Despite statistically powered studies being needed, it is reasonable to conclude that COVID-19 and SARS-CoV-2-associated endothelialitis likely make no exception, representing an ideal paradigmatic clinical scenario of investigation for glycocalyx-directed approaches [70,141,142,143,144]. 

## 7. Conclusions

Collectively, novel therapeutic windows and compounds offer promise for effectively combating intussusceptive angiogenesis and VEGF-overexpressed-ARDS following SARS-CoV-2 infection (Figure 1).

## Figures and Tables

**Table 1:** Clinical trial employing angiogenesis interacting agents in COVID-19 disease.

Study ID	Treatment	Condition	Subjects Enrolled (N)
NCT04305106	Bevacizumab 7.5 mg/kg	COVID-19	140
NCT04275414	Bevacizumab 500 mg	COVID-19	27
NCT04344782	Bevacizumab 7.5 mg/kg	COVID-19	130
NCT04329650	Siltuximab 11 mg/kg	COVID-19	20
NCT04822818	Bevacizumab 7.5 mg/kg	COVID-19	174
NCT04540926	Cyclosporine at a dose of 1–2 mg/kg/day, for 7 days	COVID-19 Pneumonia	200
NCT04412785	9 mg/kg/day oral divided q12 h, For IV 3 mg/kg/day continuous IV infusion for up to 14 days, as tolerated	Moderate COVID-19	20
NCT04492891	Cyclosporine 2.5 mg/kg PO BID 7 days and standard of care,	COVID-19	75
NCT04392531	Cyclosporine and standard of care	COVID-19	120

## Data Availability

Not applicable.

## References

[1] Lei Y., Zhang J., Schiavon C.R., He M., Chen L., Shen H., Zhang Y., Yin Q., Cho Y., Andrade L. (2021). SARS-CoV-2 Spike Protein Impairs Endothelial Function via Downregulation of ACE 2. Circ. Res..

[2] Lisco G., De Tullio A., Stragapede A., Solimando A., Albanese F., Capobianco M., Giagulli V., Guastamacchia E., De Pergola G., Vacca A. (2021). COVID-19 and the Endocrine System: A Comprehensive Review on the Theme. J. Clin. Med..

[3] Ashraf O., Young M., Malik K.J., Cheema T. (2020). Systemic Complications of COVID-19. Crit. Care Nurs. Q..

[4] Iadecola C., Anrather J., Kamel H. (2020). Effects of COVID-19 on the Nervous System. Cell.

[5] Ackermann M., Verleden S.E., Kuehnel M., Haverich A., Welte T., Laenger F., Vanstapel A., Werlein C., Stark H., Tzankov A. (2020). Pulmonary Vascular Endothelialitis, Thrombosis, and Angiogenesis in COVID-19. N. Engl. J. Med..

[6] Lan J., Ge J., Yu J., Shan S., Zhou H., Fan S., Zhang Q., Shi X., Wang Q., Zhang L. (2020). Structure of the SARS-CoV-2 spike receptor-binding domain bound to the ACE2 receptor. Nature.

[7] Gupta A., Madhavan M.V., Sehgal K., Nair N., Mahajan S., Sehrawat T.S., Bikdeli B., Ahluwalia N., Ausiello J.C., Wan E.Y. (2020). Extrapulmonary manifestations of COVID-19. Nat. Med..

[8] Ellul M.A., Benjamin L., Singh B., Lant S., Michael B.D., Easton A., Kneen R., Defres S., Sejvar J., Solomon T. (2020). Neurological associations of COVID-19. Lancet Neurol..

[9] La Marca A., Busani S., Donno V., Guaraldi G., Ligabue G., Girardis M. (2020). Testicular pain as an unusual presentation of COVID-19: A brief review of SARS-CoV-2 and the testis. Reprod. Biomed. Online.

[10] Khalili M., Iranmanesh B., Mohammadi S., Aflatoonian M. (2021). Cutaneous and histopathological features of coronavirus disease 2019 in pediatrics: A review article. Dermatol. Ther..

[11] Puntmann V.O., Carerj M.L., Wieters I., Fahim M., Arendt C., Hoffmann J., Shchendrygina A., Escher F., Vasa-Nicotera M., Zeiher A.M. (2020). Outcomes of Cardiovascular Magnetic Resonance Imaging in Patients Recently Recovered from Coronavirus Disease 2019 (COVID-19). JAMA Cardiol..

[12] Inciardi R.M., Lupi L., Zaccone G., Italia L., Raffo M., Tomasoni D., Cani D.S., Cerini M., Farina D., Gavazzi E. (2020). Cardiac Involvement in a Patient with Coronavirus Disease 2019 (COVID-19). JAMA Cardiol..

[13] Lindner D., Fitzek A., Bräuninger H., Aleshcheva G., Edler C., Meissner K., Scherschel K., Kirchhof P., Escher F., Schultheiss H.-P. (2020). Association of Cardiac Infection With SARS-CoV-2 in Confirmed COVID-19 Autopsy Cases. JAMA Cardiol..

[14] Brann D.H., Tsukahara T., Weinreb C., Lipovsek M., Van Den Berge K., Gong B., Chance R., Macaulay I.C., Chou H.-J., Fletcher R.B. (2020). Non-Neuronal expression of SARS-CoV-2 entry genes in the olfactory system suggests mechanisms underlying COVID-19-associated anosmia. Sci. Adv..

[15] Jain U. (2020). Effect of COVID-19 on the Organs. Cureus.

[16] Xydakis M.S., Albers M.W., Holbrook E.H., Lyon D.M., Shih R.Y., A Frasnelli J., Pagenstecher A., Kupke A., Enquist L.W., Perlman S. (2021). Post-Viral effects of COVID-19 in the olfactory system and their implications. Lancet Neurol..

[17] Dell’Aquila P., Raimondo P., Racanelli V., De Luca P., De Matteis S., Pistone A., Melodia R., Crudele L., Lomazzo D., Solimando A.G. (2022). Integrated lung ultrasound score for early clinical decision-making in patients with COVID-19: Results and implications. Ultrasound J..

[18] Jonigk D., Märkl B., Helms J. (2021). COVID-19: What the clinician should know about post-mortem findings. Intensiv. Care Med..

[19] Østergaard L. (2021). SARS-CoV-2 related microvascular damage and symptoms during and after COVID-19: Consequences of capillary transit-time changes, tissue hypoxia and inflammation. Physiol. Rep..

[20] Young N.S., Gerson S.L., High K.A. (2006). Clinical Hematology.

[21] Solimando A.G., Summa S.D., Vacca A., Ribatti D. (2020). Cancer-Associated Angiogenesis: The Endothelial Cell as a Checkpoint for Immunological Patrolling. Cancers.

[22] Ribatti D., Solimando A.G., Pezzella F. (2021). The Anti-VEGF(R) Drug Discovery Legacy: Improving Attrition Rates by Breaking the Vicious Cycle of Angiogenesis in Cancer. Cancers.

[23] Carmeliet P., Jain R.K. (2011). Molecular mechanisms and clinical applications of angiogenesis. Nature.

[24] Dudley A.C. (2012). Tumor Endothelial Cells. Cold Spring Harb. Perspect. Med..

[25] Garcia R.G., Garcia M.C.G., De La Fuente A.L. (1989). Impetigo Herpetiformis: Response to Steroids and Etretinate. Int. J. Dermatol..

[26] Galley H.F., Webster N.R. (2004). Physiology of the endothelium. Br. J. Anaesth..

[27] Van Hinsbergh V.W.M. (2012). Endothelium—Role in regulation of coagulation and inflammation. Semin. Immunopathol..

[28] Sun H.-J., Wu Z.-Y., Nie X.-W., Bian J.-S. (2019). Role of Endothelial Dysfunction in Cardiovascular Diseases: The Link Between Inflammation and Hydrogen Sulfide. Front. Pharmacol..

[29] Jridi I., Catacchio I., Majdoub H., Shahbazzadeh D., El Ayeb M., Frassanito M.A., Solimando A.G., Ribatti D., Vacca A., Borchani L. (2017). The small subunit of Hemilipin2, a new heterodimeric phospholipase A2 from Hemiscorpius lepturus scorpion venom, mediates the antiangiogenic effect of the whole protein. Toxicon.

[30] Van den Oever I.A.M., Raterman H.G., Nurmohamed M.T., Simsek S. (2010). Endothelial dysfunction, inflammation, and apoptosis in diabetes mellitus. Mediat. Inflamm..

[31] Wagner J.U.G., Bojkova D., Shumliakivska M., Luxán G., Nicin L., Aslan G.S., Milting H., Kandler J.D., Dendorfer A., Heumueller A.W. (2021). Increased susceptibility of human endothelial cells to infections by SARS-CoV-2 variants. Basic Res. Cardiol..

[32] Iba T., Levy J.H. (2018). Inflammation and thrombosis: Roles of neutrophils, platelets and endothelial cells and their interactions in thrombus formation during sepsis. J. Thromb. Haemost. JTH.

[33] Pober J.S., Sessa W.C. (2007). Evolving functions of endothelial cells in inflammation. Nat. Rev. Immunol..

[34] Bonaventura A., Vecchié A., Dagna L., Martinod K., Dixon D.L., Van Tassell B.W., Dentali F., Montecucco F., Massberg S., Levi M. (2021). Endothelial dysfunction and immunothrombosis as key pathogenic mechanisms in COVID-19. Nat. Rev. Immunol..

[35] Cicco S., Vacca A., Cariddi C., Carella R., Altamura G., Solimando A.G., Lauletta G., Pappagallo F., Cirulli A., Stragapede A. (2021). Imaging Evaluation of Pulmonary and Non-Ischaemic Cardiovascular Manifestations of COVID-19. Diagnostics.

[36] Dejana E., Spagnuolo R., Bazzoni G. (2001). Interendothelial junctions and their role in the control of angiogenesis, vascular permeability and leukocyte transmigration. Thromb. Haemost..

[37] Wallez Y., Huber P. (2008). Endothelial adherens and tight junctions in vascular homeostasis, inflammation and angiogenesis. Biochim. Biophys. Acta.

[38] Solimando A.G., Da Vià M.C., Leone P., Borrelli P., Croci G.A., Tabares P., Brandl A., Di Lernia G., Bianchi F.P., Tafuri S. (2021). Halting the vicious cycle within the multiple myeloma ecosystem: Blocking JAM-A on bone marrow endothelial cells restores angiogenic homeostasis and suppresses tumor progression. Haematologica.

[39] Segal S.S. (2005). Regulation of Blood Flow in the Microcirculation. Microcirculation.

[40] Takeshita Y., Ransohoff R.M. (2012). Inflammatory cell trafficking across the blood-brain barrier: Chemokine regulation and in vitro models. Immunol. Rev..

[41] Salmi M., Jalkanen S. (2005). Cell-Surface enzymes in control of leukocyte trafficking. Nat. Rev. Immunol..

[42] Karami H., Derakhshani A., Ghasemigol M., Fereidouni M., Miri-Moghaddam E., Baradaran B., Tabrizi N.J., Najafi S., Solimando A.G., Marsh L.M. (2021). Weighted Gene Co-Expression Network Analysis Combined with Machine Learning Validation to Identify Key Modules and Hub Genes Associated with SARS-CoV-2 Infection. J. Clin. Med..

[43] Becker R.C. (2020). COVID-19-Associated vasculitis and vasculopathy. J. Thromb. Thrombolysis.

[44] Knight S.R., Ho A., Pius R., Buchan I., Carson G., Drake T.M., Dunning J., Fairfield C.J., Gamble C., Green C.A. (2020). Risk stratification of patients admitted to hospital with COVID-19 using the ISARIC WHO Clinical Characterisation Protocol: Development and validation of the 4C Mortality Score. BMJ.

[45] Solimando A.G., Susca N., Borrelli P., Prete M., Lauletta G., Pappagallo F., Buono R., Inglese G., Forina B.M., Bochicchio D. (2020). Short-Term Variations in Neutrophil-to-Lymphocyte and Urea-to-Creatinine Ratios Anticipate Intensive Care Unit Admission of COVID-19 Patients in the Emergency Department. Front. Med..

[46] Ter Meulen J., Bakker A.B.H., Brink E.N.V.D., Weverling G.J., Martina B.E.E., Haagmans B.L., Kuiken T., de Kruif J., Preiser W., Spaan W. (2004). Human monoclonal antibody as prophylaxis for SARS coronavirus infection in ferrets. Lancet.

[47] Bavaro D., Diella L., Solimando A., Cicco S., Buonamico E., Stasi C., Ciannarella M., Marrone M., Carpagnano F., Resta O. (2022). Bamlanivimab and Etesevimab administered in an outpatient setting for SARS-CoV-2 infection. Pathog. Glob. Health.

[48] Gandhi R.T., Malani P.N., Del Rio C. (2022). COVID-19 Therapeutics for Nonhospitalized Patients. JAMA.

[49] Tian W., Jiang W., Yao J., Nicholson C.J., Li R., Sigurslid H., Wooster L., Rotter J.I., Guo X., Malhotra R. (2020). Predictors of mortality in hospitalized COVID-19 patients: A systematic review and meta-analysis. J. Med. Virol..

[50] Cavalli E., Bramanti A., Ciurleo R., Tchorbanov A.I., Giordano A., Fagone P., Belizna C., Bramanti P., Shoenfeld Y., Nicoletti F. (2020). Entangling COVID-19 associated thrombosis into a secondary antiphospholipid antibody syndrome: Diagnostic and therapeutic perspectives (Review). Int. J. Mol. Med..

[51] Zhang Y., Xiao M., Zhang S., Xia P., Cao W., Jiang W., Chen H., Ding X., Zhao H., Zhang H. (2020). Coagulopathy and Antiphospholipid Antibodies in Patients with COVID-19. N. Engl. J. Med..

[52] Escher R., Breakey N., Lämmle B. (2020). Severe COVID-19 infection associated with endothelial activation. Thromb. Res..

[53] Harzallah I., Debliquis A., Drénou B. (2020). Lupus anticoagulant is frequent in patients with COVID-19. J. Thromb. Haemost..

[54] Gazzaruso C., Stella N.C., Mariani G., Nai C., Coppola A., Naldani D., Gallotti P. (2020). High prevalence of antinuclear antibodies and lupus anticoagulant in patients hospitalized for SARS-CoV2 pneumonia. Clin. Rheumatol..

[55] Knight J.S., Caricchio R., Casanova J.-L., Combes A.J., Diamond B., Fox S.E., Hanauer D.A., James J.A., Kanthi Y., Ladd V. (2021). The intersection of COVID-19 and autoimmunity. J. Clin. Investig..

[56] Woodruff M.C., Ramonell R.P., Nguyen D.C., Cashman K.S., Saini A.S., Haddad N.S., Ley A.M., Kyu S., Howell J.C., Ozturk T. (2020). Extrafollicular B cell responses correlate with neutralizing antibodies and morbidity in COVID-19. Nat. Immunol..

[57] Wang E.Y., Mao T., Klein J., Dai Y., Huck J.D., Jaycox J.R., Liu F., Zhou T., Israelow B., Wong P. (2021). Diverse Functional Autoantibodies in Patients with COVID-19. medRxiv.

[58] Zuo Y., Estes S.K., Ali R.A., Gandhi A.A., Yalavarthi S., Shi H., Sule G., Gockman K., Madison J.A., Zuo M. (2020). Prothrombotic autoantibodies in serum from patients hospitalized with COVID-19. Sci. Transl. Med..

[59] Hollerbach A., Müller-Calleja N., Pedrosa D., Canisius A., Sprinzl M.F., Falter T., Rossmann H., Bodenstein M., Werner C., Sagoschen I. (2021). Pathogenic lipid-binding antiphospholipid antibodies are associated with severity of COVID-19. J. Thromb. Haemost. JTH.

[60] Franchini M., Marano G., Cruciani M., Mengoli C., Pati I., Masiello F., Veropalumbo E., Pupella S., Vaglio S., Liumbruno G.M. (2020). COVID-19-Associated coagulopathy. Diagnosis.

[61] Becker R.C. (2020). COVID-19 update: COVID-19-Associated coagulopathy. J. Thromb. Thrombolysis.

[62] Zaid Y., Puhm F., Allaeys I., Naya A., Oudghiri M., Khalki L., Limami Y., Zaid N., Sadki K., Ben El Haj R. (2020). Platelets Can Associate With SARS-CoV-2 RNA and Are Hyperactivated in COVID-19. Circ. Res..

[63] Sriram K., Insel P.A. (2021). Inflammation and thrombosis in COVID-19 pathophysiology: Proteinase-Activated and purinergic receptors as drivers and candidate therapeutic targets. Physiol. Rev..

[64] Franciosi M.L.M., Lima M.D.M., Schetinger M.R.C., Cardoso A.M. (2021). Possible role of purinergic signaling in COVID-19. Mol. Cell. Biochem..

[65] Battina H.L., Alentado V.J., Srour E.F., Moliterno A.R., Kacena M.A. (2021). Interaction of the inflammatory response and megakaryocytes in COVID-19 infection. Exp. Hematol..

[66] O’Sullivan J.M., Mc Gonagle D., E Ward S., Preston R.J.S., O’Donnell J.S. (2020). Endothelial cells orchestrate COVID-19 coagulopathy. Lancet Haematol..

[67] Pandey A., Nikam A.N., Shreya A.B., Mutalik S.P., Gopalan D., Kulkarni S., Padya B.S., Fernandes G., Mutalik S., Prassl R. (2020). Potential therapeutic targets for combating SARS-CoV-2: Drug repurposing, clinical trials and recent advancements. Life Sci..

[68] Libby P., Lüscher T. (2020). COVID-19 is, in the end, an endothelial disease. Eur. Heart J..

[69] Varga Z., Flammer A.J., Steiger P., Haberecker M., Andermatt R., Zinkernagel A.S., Mehra M.R., Schuepbach R.A., Ruschitzka F., Moch H. (2020). Endothelial cell infection and endotheliitis in COVID-19. Lancet.

[70] Stahl K., Gronski P.A., Kiyan Y., Seeliger B., Bertram A., Pape T., Welte T., Hoeper M.M., Haller H., David S. (2020). Injury to the Endothelial Glycocalyx in Critically Ill Patients with COVID-19. Am. J. Respir. Crit. Care Med..

[71] Schmaier A.A., Hurtado G.M.P., Manickas-Hill Z.J., Sack K.D., Chen S.M., Bhambhani V., Quadir J., Nath A.K., Collier A.-R.Y., Ngo D. (2021). Tie2 activation protects against prothrombotic endothelial dysfunction in COVID-19. JCI Insight.

[72] Manne B.K., Denorme F., Middleton E.A., Portier I., Rowley J.W., Stubben C., Petrey A.C., Tolley N.D., Guo L., Cody M. (2020). Platelet gene expression and function in patients with COVID-19. Blood.

[73] Taus F., Salvagno G., Canè S., Fava C., Mazzaferri F., Carrara E., Petrova V., Barouni R.M., Dima F., Dalbeni A. (2020). Platelets Promote Thromboinflammation in SARS-CoV-2 Pneumonia. Arterioscler. Thromb. Vasc. Biol..

[74] Brinkmann V., Reichard U., Goosmann C., Fauler B., Uhlemann Y., Weiss D.S., Weinrauch Y., Zychlinsky A. (2004). Neutrophil extracellular traps kill bacteria. Science.

[75] Zuo Y., Yalavarthi S., Navaz S.A., Hoy C.K., Harbaugh A., Gockman K., Zuo M., Madison J.A., Shi H., Kanthi Y. (2021). Autoantibodies stabilize neutrophil extracellular traps in COVID-19. JCI Insight.

[76] Middleton E.A., He X.-Y., Denorme F., Campbell R.A., Ng D., Salvatore S.P., Mostyka M., Baxter-Stoltzfus A., Borczuk A.C., Loda M. (2020). Neutrophil extracellular traps contribute to immunothrombosis in COVID-19 acute respiratory distress syndrome. Blood.

[77] Skendros P., Mitsios A., Chrysanthopoulou A., Mastellos D.C., Metallidis S., Rafailidis P., Ntinopoulou M., Sertaridou E., Tsironidou V., Tsigalou C. (2020). Complement and tissue factor–enriched neutrophil extracellular traps are key drivers in COVID-19 immunothrombosis. J. Clin. Investig..

[78] Holter J.C., Pischke S.E., de Boer E., Lind A., Jenum S., Holten A.R., Tonby K., Barratt-Due A., Sokolova M., Schjalm C. (2020). Systemic complement activation is associated with respiratory failure in COVID-19 hospitalized patients. Proc. Natl. Acad. Sci. USA.

[79] Magro C., Mulvey J.J., Berlin D., Nuovo G., Salvatore S., Harp J., Baxter-Stoltzfus A., Laurence J. (2020). Complement associated microvascular injury and thrombosis in the pathogenesis of severe COVID-19 infection: A report of five cases. Transl. Res..

[80] Cugno M., Meroni P.L., Gualtierotti R., Griffini S., Grovetti E., Torri A., Lonati P., Grossi C., Borghi M.O., Novembrino C. (2021). Complement activation and endothelial perturbation parallel COVID-19 severity and activity. J. Autoimmun..

[81] Shaw R.J., Bradbury C., Abrams S.T., Wang G., Toh C. (2021). COVID-19 and immunothrombosis: Emerging understanding and clinical management. Br. J. Haematol..

[82] Rahaman M.M., Li C., Yao Y., Kulwa F., Rahman M.A., Wang Q., Qi S., Kong F., Zhu X., Zhao X. (2020). Identification of COVID-19 samples from chest X-Ray images using deep learning: A comparison of transfer learning approaches. J. X-ray Sci. Technol..

[83] Pontone G., Scafuri S., Mancini M.E., Agalbato C., Guglielmo M., Baggiano A., Muscogiuri G., Fusini L., Andreini D., Mushtaq S. (2021). Role of computed tomography in COVID-19. J. Cardiovasc. Comput. Tomogr..

[84] Zhang K., Liu X., Shen J., Li Z., Sang Y., Wu X., Zha Y., Liang W., Wang C., Wang K. (2020). Clinically Applicable AI System for Accurate Diagnosis, Quantitative Measurements, and Prognosis of COVID-19 Pneumonia Using Computed Tomography. Cell.

[85] Wasilewski P., Mruk B., Mazur S., Półtorak-Szymczak G., Sklinda K., Walecki J. (2020). COVID-19 severity scoring systems in radiological imaging—A review. Pol. J. Radiol..

[86] Shi H., Han X., Jiang N., Cao Y., Alwalid O., Gu J., Fan Y., Zheng C. (2020). Radiological findings from 81 patients with COVID-19 pneumonia in Wuhan, China: A descriptive study. Lancet Infect. Dis..

[87] Caramaschi S., Kapp M.E., Miller S.E., Eisenberg R., Johnson J., Epperly G., Maiorana A., Silvestri G., Giannico G.A. (2021). Giannico. Histopathological findings and clinicopathologic correlation in COVID-19: A systematic review. Mod. Pathol..

[88] Ciceri F., Beretta L., Scandroglio A.M., Colombo S., Landoni G., Ruggeri A., Peccatori J., D’Angelo A., De Cobelli F., Rovere-Querini P. (2020). Microvascular COVID-19 lung vessels obstructive thromboinflammatory syndrome (MicroCLOTS): An atypical acute respiratory distress syndrome working hypothesis. Crit. Care Resusc. J. Australas. Acad. Crit. Care Med..

[89] Barisione E., Grillo F., Ball L., Bianchi R., Grosso M., Morbini P., Pelosi P., Patroniti N.A., De Lucia A., Orengo G. (2021). Fibrotic progression and radiologic correlation in matched lung samples from COVID-19 post-mortems. Virchows Arch. Int. J. Pathol..

[90] Colmenero I., Santonja C., Alonso-Riaño M., Noguera-Morel L., Hernández-Martín A., Andina D., Wiesner T., Rodríguez-Peralto J., Requena L., Torrelo A. (2020). SARS-CoV-2 endothelial infection causes COVID-19 chilblains: Histopathological, immunohistochemical and ultrastructural study of seven paediatric cases. Br. J. Dermatol..

[91] Liu F., Han K., Blair R., Kenst K., Qin Z., Upcin B., Wörsdörfer P., Midkiff C.C., Mudd J., Belyaeva E. (2021). SARS-CoV-2 Infects Endothelial Cells In Vivo and In Vitro. Front. Cell. Infect. Microbiol..

[92] Schimmel L., Chew K.Y., Stocks C.J., E Yordanov T., Essebier P., Kulasinghe A., Monkman J., Miggiolaro A.F.R.D.S., Cooper C., de Noronha L. (2021). Endothelial cells are not productively infected by SARS-CoV-2. Clin. Transl. Immunol..

[93] Meini S., Giani T., Tascini C. (2020). Intussusceptive angiogenesis in COVID-19: Hypothesis on the significance and focus on the possible role of FGF2. Mol. Biol. Rep..

[94] Deigendesch N., Sironi L., Kutza M., Wischnewski S., Fuchs V., Hench J., Frank A., Nienhold R., Mertz K.D., Cathomas G. (2020). Correlates of critical illness-related encephalopathy predominate postmortem COVID-19 neuropathology. Acta Neuropathol..

[95] Liu Y., Cox S.R., Morita T., Kourembanas S. (1995). Hypoxia Regulates Vascular Endothelial Growth Factor Gene Expression in Endothelial Cells: Identification of a 5′ Enhancer. Circ. Res..

[96] Theofilis P., Sagris M., Oikonomou E., Antonopoulos A.S., Siasos G., Tsioufis C., Tousoulis D. (2021). Inflammatory Mechanisms Contributing to Endothelial Dysfunction. Biomedicines.

[97] Ackermann M., Mentzer S.J., Kolb M., Jonigk D. (2020). Inflammation and intussusceptive angiogenesis in COVID-19: Everything in and out of flow. Eur. Respir. J..

[98] Madureira G., Soares R. (2021). The misunderstood link between SARS-CoV-2 and angiogenesis. A narrative review. Pulmonology.

[99] Djonov V., Schmid M., Tschanz S.A., Burri P.H. (2000). Intussusceptive angiogenesis: Its role in embryonic vascular network formation. Circ. Res..

[100] Djonov V., Baum O., Burri P.H. (2003). Vascular remodeling by intussusceptive angiogenesis. Cell Tissue Res..

[101] Burri P.H., Hlushchuk R., Djonov V. (2004). Intussusceptive angiogenesis: Its emergence, its characteristics, and its significance. Dev. Dyn. Off. Publ. Am. Assoc. Anat..

[102] André H., Tunik S., Aronsson M., Kvanta A. (2015). Hypoxia-Inducible Factor-1α Is Associated with Sprouting Angiogenesis in the Murine Laser-Induced Choroidal Neovascularization Model. Investig. Opthalmol. Vis. Sci..

[103] Hlushchuk R., Riesterer O., Baum O., Wood J., Gruber G., Pruschy M., Djonov V. (2008). Tumor Recovery by Angiogenic Switch from Sprouting to Intussusceptive Angiogenesis after Treatment with PTK787/ZK222584 or Ionizing Radiation. Am. J. Pathol..

[104] Siddiqi H.K., Libby P., Ridker P.M. (2021). COVID-19—A vascular disease. Trends Cardiovasc. Med..

[105] Rhodes R.H., Love G.L., Da Silva Lameira F., Sadough M.S., Fox S.E., Heide R.S.V. (2021). Acute Endotheliitis (Type 3 Hypersensitivity Vasculitis) in Ten COVID-19 Autopsy Brains. Pathology.

[106] Da Vià M.C., Solimando A.G., Garitano-Trojaola A., Barrio S., Munawar U., Strifler S., Haertle L., Rhodes N., Teufel E., Vogt C. (2019). CIC Mutation as a Molecular Mechanism of Acquired Resistance to Combined BRAF-MEK Inhibition in Extramedullary Multiple Myeloma with Central Nervous System Involvement. Oncologist.

[107] Ramlall V., Thangaraj P.M., Meydan C., Foox J., Butler D., Kim J., May B., De Freitas J.K., Glicksberg B.S., Mason C.E. (2020). Immune complement and coagulation dysfunction in adverse outcomes of SARS-CoV-2 infection. Nat. Med..

[108] Smadja D.M., Mentzer S.J., Fontenay M., Laffan M.A., Ackermann M., Helms J., Jonigk D., Chocron R., Pier G.B., Gendron N. (2021). COVID-19 is a systemic vascular hemopathy: Insight for mechanistic and clinical aspects. Angiogenesis.

[109] Menter T., Haslbauer J.D., Nienhold R., Savic S., Hopfer H., Deigendesch N., Frank S., Turek D., Willi N., Pargger H. (2020). Post-Mortem examination of COVID19 patients reveals diffuse alveolar damage with severe capillary congestion and variegated findings of lungs and other organs suggesting vascular dysfunction. Histopathology.

[110] Schaller T., Hirschbühl K., Burkhardt K., Braun G., Trepel M., Märkl B., Claus R. (2020). Postmortem Examination of Patients With COVID-19. JAMA.

[111] Lax S.F., Skok K., Zechner P., Kessler H.H., Kaufmann N., Koelblinger C., Vander K., Bargfrieder U., Trauner M. (2020). Pulmonary Arterial Thrombosis in COVID-19 with Fatal Outcome: Results From a Prospective, Single-Center, Clinicopathologic Case Series. Ann. Intern. Med..

[112] Fox S.E., Akmatbekov A., Harbert J.L., Li G., Brown J.Q., Heide R.S.V. (2020). Pulmonary and cardiac pathology in African American patients with COVID-19: An autopsy series from New Orleans. Lancet Respir. Med..

[113] Carsana L., Sonzogni A., Nasr A., Rossi R.S., Pellegrinelli A., Zerbi P., Rech R., Colombo R., Antinori S., Corbellino M. (2020). Pulmonary post-mortem findings in a series of COVID-19 cases from northern Italy: A two-centre descriptive study. Lancet Infect. Dis..

[114] Mentzer S.J., Konerding M.A. (2014). Intussusceptive angiogenesis: Expansion and remodeling of microvascular networks. Angiogenesis.

[115] Burri P.H., Djonov V. (2002). Intussusceptive angiogenesis––The alternative to capillary sprouting. Mol. Asp. Med..

[116] Kolte D., McClung J.A., Aronow W.S. (2016). Vasculogenesis and Angiogenesis. Translational Research in Coronary Artery Disease.

[117] Kong Y., Han J., Wu X., Zeng H., Liu J., Zhang H. (2020). VEGF-D: A novel biomarker for detection of COVID-19 progression. Crit. Care.

[118] Yin X.-X., Zheng X.-R., Peng W., Wu M.-L., Mao X.-Y. (2020). Vascular Endothelial Growth Factor (VEGF) as a Vital Target for Brain Inflammation during the COVID-19 Outbreak. ACS Chem. Neurosci..

[119] Landecho M.F., Yuste J.R., Gándara E., Sunsundegui P., Quiroga J., Alcaide A.B., García-Layana A. (2021). COVID-19 retinal microangiopathy as an in vivo biomarker of systemic vascular disease?. J. Intern. Med..

[120] Erickson M.A., Rhea E.M., Knopp R.C., Banks W.A. (2021). Interactions of SARS-CoV-2 with the Blood–Brain Barrier. Int. J. Mol. Sci..

[121] Barratt S., Medford A.R., Millar A.B. (2014). Vascular endothelial growth factor in acute lung injury and acute respiratory distress syndrome. Respir. Int. Rev. Thorac. Dis..

[122] Moutal A., Martin L.F., Boinon L., Gomez K., Ran D., Zhou Y., Stratton H.J., Cai S., Luo S., Gonzalez K.B. (2020). SARS-CoV-2 spike protein co-opts VEGF-A/neuropilin-1 receptor signaling to induce analgesia. Pain.

[123] Smadja D.M., Philippe A., Bory O., Gendron N., Beauvais A., Gruest M., Peron N., Khider L., Guerin C.L., Goudot G. (2021). Placental growth factor level in plasma predicts COVID-19 severity and in-hospital mortality. J. Thromb. Haemost. JTH.

[124] Pine A.B., Meizlish M.L., Goshua G., Chang C.H., Zhang H., Bishai J., Bahel P., Patel A., Gbyli R., Kwan J.M. (2020). Circulating markers of angiogenesis and endotheliopathy in COVID-19. Pulm. Circ..

[125] Rovas A., Osiaevi I., Buscher K., Sackarnd J., Tepasse P.-R., Fobker M., Kühn J., Braune S., Göbel U., Thölking G. (2021). Microvascular dysfunction in COVID-19: The MYSTIC study. Angiogenesis.

[126] Mazzoni A., Salvati L., Maggi L., Capone M., Vanni A., Spinicci M., Mencarini J., Caporale R., Peruzzi B., Antonelli A. (2020). Impaired immune cell cytotoxicity in severe COVID-19 is IL-6 dependent. J. Clin. Investig..

[127] Gopinathan G., Milagre C., Pearce O.M., Reynolds L.E., Hodivala-Dilke K., Leinster D.A., Zhong H., Hollingsworth R.E., Thompson R., Whiteford J.R. (2015). Interleukin-6 Stimulates Defective Angiogenesis. Cancer Res..

[128] Sahebnasagh A., Avan R., Saghafi F., Mojtahedzadeh M., Sadremomtaz A., Arasteh O., Tanzifi A., Faramarzi F., Negarandeh R., Safdari M. (2020). Pharmacological treatments of COVID-19. Pharmacol. Rep. PR.

[129] Smadja D.M., Guerin C.L., Chocron R., Yatim N., Boussier J., Gendron N., Khider L., Hadjadj J., Goudot G., Debuc B. (2020). Angiopoietin-2 as a marker of endothelial activation is a good predictor factor for intensive care unit admission of COVID-19 patients. Angiogenesis.

[130] Tanabe K., Maeshima Y., Sato Y., Wada J. (2017). Antiangiogenic Therapy for Diabetic Nephropathy. BioMed Res. Int..

[131] Argentiero A., Solimando A.G., Krebs M., Leone P., Susca N., Brunetti O., Racanelli V., Vacca A., Silvestris N. (2020). Anti-Angiogenesis and Immunotherapy: Novel Paradigms to Envision Tailored Approaches in Renal Cell-Carcinoma. J. Clin. Med..

[132] Antonio G., Oronzo B., Vito L., Angela C., Antonel-La A., Roberto C., Giovanni S.A., Antonella L. (2020). Immune system and bone microenvironment: Rationale for targeted cancer therapies. Oncotarget.

[133] Derakhshani A., Hashemzadeh S., Asadzadeh Z., Shadbad M., Rasibonab F., Safarpour H., Jafarlou V., Solimando A., Racanelli V., Singh P. (2021). Cytotoxic T-Lymphocyte Antigen-4 in Colorectal Cancer: Another Therapeutic Side of Capecitabine. Cancers.

[134] Martin A., Komada M.R., Sane D.C. (2003). Abnormal angiogenesis in diabetes mellitus. Med. Res. Rev..

[135] Pang J., Xu F., Aondio G., Li Y., Fumagalli A., Lu M., Valmadre G., Wei J., Bian Y., Canesi M. (2021). Efficacy and tolerability of bevacizumab in patients with severe COVID-19. Nat. Commun..

[136] Lever R., Rose M.J., McKenzie E.A., Page C.P. (2014). Heparanase Induces Inflammatory Cell Recruitment in Vivo by Promoting Adhesion to Vascular Endothelium. Am. J. Physiol. Cell. Physiol..

[137] LaRivière W.B., Schmidt E.P. (2018). The Pulmonary Endothelial Glycocalyx in ARDS: A Critical Role for Heparan Sulfate. Current Topics in Membranes.

[138] Buijsers B., Yanginlar C., de Nooijer A., Grondman I., Maciej-Hulme M.L., Jonkman I., Janssen N.A.F., Rother N., de Graaf M., Pickkers P. (2020). Increased Plasma Heparanase Activity in COVID-19 Patients. Front. Immunol..

[139] Lukasz A., Hillgruber C., Oberleithner H., Kusche-Vihrog K., Pavenstädt H., Rovas A., Hesse B., Goerge T., Kümpers P. (2017). Endothelial Glycocalyx Breakdown Is Mediated by Angiopoietin-2. Cardiovasc. Res..

[140] Han S., Lee S.-J., Kim K.E., Lee H.S., Oh N., Park I., Ko E., Oh S.J., Lee Y.-S., Kim D. (2016). Amelioration of Sepsis by TIE2 Activation-Induced Vascular Protection. Sci. Transl. Med..

[141] Yamaoka-Tojo M. (2020). Endothelial Glycocalyx Damage as a Systemic Inflammatory Microvascular Endotheliopathy in COVID-19. Biomed. J..

[142] Drost C.C., Rovas A., Osiaevi I., Rauen M., van der Vlag J., Buijsers B., Salmenov R., Lukasz A., Pavenstädt H., Linke W.A. (2022). Heparanase Is a Putative Mediator of Endothelial Glycocalyx Damage in COVID-19—A Proof-of-Concept Study. Front. Immunol..

[143] Potje S.R., Costa T.J., Fraga-Silva T.F.C., Martins R.B., Benatti M.N., Almado C.E.L., de Sá K.S.G., Bonato V.L.D., Arruda E., Louzada-Junior P. (2021). Heparin Prevents in Vitro Glycocalyx Shedding Induced by Plasma from COVID-19 Patients. Life Sci..

[144] Okada H., Yoshida S., Hara A., Ogura S., Tomita H. (2021). Vascular Endothelial Injury Exacerbates Coronavirus Disease 2019: The Role of Endothelial Glycocalyx Protection. Microcirculation.

